# Assessment of the Attitudes, Beliefs, and Potential Effects of Nutritional Phytoestrogenic Plant Consumption on Women’s Health in Saudi Arabia

**DOI:** 10.7759/cureus.40918

**Published:** 2023-06-25

**Authors:** Halima Elagib, Shaden S Alshammari, Wefaq K Alsadoon, Aqeela Zahra

**Affiliations:** 1 Pharmacology, Faculty of Pharmacy, Omdurman Islamic University, Omdurman, SDN; 2 Pharmacology, College of Medicine, University of Hail College of Medicine, Hail, SAU; 3 Medicine and Surgery, University of Hail College of Medicine, Hail, SAU; 4 Community and Family Medicine, Leicester Medical School, University of Leicester, Leicester, GBR

**Keywords:** potential, assessment, health, women, beliefs, attitudes, tumor, consumption, herbs, phytoestrogen

## Abstract

Background and objective: Phytoestrogens are plant-derived endocrine-disrupting chemicals known as estrogen-like compounds with health and preventive benefits. This study aimed to assess Saudi Arabian women's beliefs and attitudes toward phytoestrogen-rich plant consumption and its association with hormone-sensitive tumors and diseases.

Methods: A cross-sectional study was carried out between November 2022 and May 2023 in Saudi Arabia using an online self-administered questionnaire created via Google Forms (Appendix). The collected data were extracted, coded, and analyzed using the IBM Corp. Released 2012. IBM SPSS Statistics for Windows, Version 21.0. Armonk, NY: IBM Corp.

Results: The study included 702 females aged 18 years and older. 61.6% (n=432) of the participants consumed Trigonella foenum-graecum (fenugreek) and Foeniculum vulgare (fennel), whereas 45.7% (n=321) consumed soy products and flaxseeds. In addition, 44.7% (n=191) were diagnosed with breast tumors and other breast diseases. The association between the occurrence of breast conditions and the consumption of all the different types of phytoestrogen-rich plants in this study was significant, with a p-value of <0.001. (41.7%, n=187) diagnosed with uterine and ovarian-related conditions, the association with the consumption of fenugreek and fennel was significant with a p-value of <0.001, but for soy products and flaxseed consumption, the association was not significant with a p-value of 0.368. Regarding the consumer's attitudes and duration of consumption, most of them (41.0%, n=288) consume phytoestrogen-rich herbs and plants about every month during menstruation, and 55% (n=386) consumed phytoestrogen-rich plants a long time ago (for many years). About the purpose of consumption, 62.1% (n=436) answered to reduce pain and symptoms associated with menstruation. The majority of the participants (36.3%, n=255) have poor knowledge and don’t know about the high estrogen levels' impact on their health.

Conclusion: The study showed that there is a possible association between the consumption of some types of phytoestrogen-rich plants and the occurrence of several hormone-related tumors and diseases. Further studies are needed to evaluate the possible effects in consideration of the co-founding factors.

## Introduction

For many decades, it has been widely acknowledged that diet significantly impacts human health. The consumption of specific dietary components can either enhance or harm human health depending on the nutritional constituents present in the diet; one of these components is phytoestrogens.

Phytoestrogens are naturally occurring non-steroidal estrogens found in various plant species and foodstuffs, such as vegetables, fruits, and herbs. They comprise a class of widely dispersed nutritional compounds that have different structural compounds, including isoflavones, lignans, and stilbenes [1]. Phytoestrogens share strong similarities with mammalian estrogen, estradiol, and they bind to estrogen receptors α and β, with a preference for the more recently identified β estrogen receptor [2]. These estrogen receptor subtypes play multiple roles in cancer biology, therapy, and gene regulation. Estrogen receptor alpha enhances cell proliferation, which is important for growth and tissue maintenance, but it may also play a role in the unlimited growth of estrogen receptors α-dependent breast tumors. Conversely, estrogen receptor β have the opposite role, promoting apoptosis.

The activity of these estrogen receptors depends on the ability of chemical compounds consumed in the diet to interact with them [1-3]. Increased consumption of phytoestrogen herbs among females has been observed in our society. Some women assume that consuming these herbs would help them increase their estrogen levels and acquire more feminine features. Others believe that consuming these herbs could enhance their health in various aspects. However, we wonder if consuming phytoestrogen plants could do the opposite and harm health, leading to estrogen-dependent tumors and other hormone-related diseases, especially in individuals with genetic susceptibility. Therefore, we decided to conduct research to find answers to our questions. We conducted research on various hormone-dependent health conditions, as written below, in addition to ovarian cysts and uterine malignant tumors.

Hormone-dependent diseases

Fibroadenoma is a benign, well-circumscribed, painless, and unilateral breast condition that is hormonally sensitive and affects females between 14 and 35 years old. Its incidence expands during pregnancy and declines throughout menopause. An increase in estrogen levels contributes to the development of fibroadenoma [4-5]. Phyllodes tumors are rare fibroepithelial tumors classified histologically into benign, borderline, and malignant tumors. Benign phyllodes tumors appear more frequently than the other types [6].

Breast cancer is a heterogeneous disease, and gene-expression profiling identified two main groups based on estrogen receptor (ER) expression: ER-positive and ER-negative. Tumors that are ER-positive have a stronger association with hormone-related factors than tumors that are ER-negative [7]. Some studies have revealed an association between benign breast lesions developing into invasive carcinoma. Moreover, exposure to estrogen from external sources for a long period of time is a risk factor for breast cancer [4]. Breast cysts are part of the large category of fibrocystic diseases of the breast and represent one of the most common causes of breast masses in females. This disease involves a wide range of fibrous and cystic changes in breast tissue [8].

Fibroids are the most common benign tumors of the myometrium, which is the smooth muscle of the uterus in females. It mainly affects premenopausal women. Studies show that estradiol and progesterone, ovarian steroids, promote the development of fibroids and that the size of fibroids frequently decreases after menopause when those hormone levels fall. As a result, the fibroid is known to be extremely sensitive to the effects of steroid hormones in medicine [4,9,10]. This study aimed to assess the contribution of traditional herb consumption to the manifestations of gynecological health problems to raise awareness amongst females about their health and build a better understanding of their choices in daily consumption.

## Materials and methods

A descriptive cross-sectional study was conducted among women aged 18 years and older in Saudi Arabia between November 2022 and May 2023. An online survey form (Appendix) was circulated through social media (WhatsApp, Telegram, Twitter, Instagram) and among clients who visited gynecology and oncology clinics in several health centers in Ha’il City, Saudi Arabia.

The female population in Saudi Arabia will be 14,747,165 million in mid-2021, according to the General Authority for Statistics [11]. The inclusion criteria of the present study targeted females aged 18 years and older, females under 18 years were excluded. Regarding the sample size, the female population is approximately 10,633,642 million, which represents 33% of the total population. The sample size is calculated by keeping the confidence interval at 95% and the margin of error at 5%. The minimum required sample size is 385. However, data was collected from 702 participants to account for any bias or incompleteness. Based on exclusion criteria, any data entry errors and incomplete forms are excluded.

The questionnaire was self-administered and composed of four sections: The first section was about the sociodemographic data, which included information about age, nationality, marital status, and place of residence. In the second section, designed to obtain information about their knowledge and consumption of phytoestrogen derivatives in general, we did not specify asking about specific preparations of the mentioned plants, and all forms of the plants are accepted in this study. The third part was about attitude as well as the purpose of consumption, such as duration and time. The fourth section was about the diagnosis of hormone-sensitive tumors. Lastly, we explained what estrogen is, its role in the human body, and the fact that there are similar compounds to estrogen found in various plants and herbs to ensure that the participants would fill out the questionnaire with good knowledge about the idea of this study. The questionnaire design was guided through a pilot study with 702 participants to meet our objectives. 

The data were analyzed using IBM Corp. Released 2012. IBM SPSS Statistics for Windows, Version 21.0. Armonk, NY: IBM Corp. Descriptive statistics and analysis of the variables regarding the sociodemographic data were carried out. Chi-square tests were performed to evaluate the association between phytoestrogen plant derivative consumption and the presence of hormone-sensitive tumors and diseases by using bivariate analysis. The statistical results were verified using a significant level of p< 0.05. The results are presented in tables and figures.

Ethical consideration

The study was reviewed and approved by the Research Ethics Committee (REC) at the University of Ha’il (approval no. H-2022-391). A consent form was taken from all the participants at the beginning of the questionnaires.

## Results

A total of 702 participants completed the questionnaire. The majority of respondents' age group reported was 18-25 years old (n=335, 47.7%), with a mean of 26.12 years old and a standard deviation of 1.26. Most participants identified as Saudi (n=661, 94.2%) and resided in the Northern region (n=223, 31.8%). In terms of marital status, single was the most frequently observed category (n=359, 51.1%), as shown in Table 1.

**Table 1:** Demographic characteristics of participants (N=702)

Characteristics	N	%
Age		
18-25 years	335	47.7
26-35 years	101	14.4
36-45 years	148	21.1
46-55 years	87	12.4
56-65 years	28	4.0
>65 years	3	0.4
Nationality		
Saudi	661	94.2
Non-Saudi	41	5.8
Region		
Central region	91	13.0
Northern region	223	31.8
Eastern region	148	21.1
Western region	191	27.2
Southern region	49	7.0
Social status		
Single	359	51.1
Married	310	44.2
Divorced	27	3.8
Widow	6	0.9

Regarding the attitude and purpose of consumption, our analysis showed that most of the participants consumed phytoestrogen-rich plants and herbs at some point in their lives (n=568, 80,9%). Some of them (n=234, 34.6%) have stopped consuming these herbs (n=386, 55.0%); they started consuming phytoestrogens (plants and herbs) a long time ago (for many years). The most common types consumed are both fenugreek and fennel (n=216, 30.8%), followed by flaxseeds (n=145, 20.7%). In terms of evaluating the duration and time of consumption, most of the consumers use these herbs and plants during their menstruation times (n=288, 41.0%), and 130 (18.5%) of participants use the herbs between long intermittent periods. Regarding the purpose of consumption, most of them (n=436; 62.1%) chose to reduce pain and symptoms associated with menstruation, as shown in Table 2.

**Table 2:** Attitude and purpose of herb consumption (N=702)

Question 1		N	%
Have you ever consumed these herbs such as: fennel, fenugreek?	No	134	19.1
	Yes	568	80.9
Question 2			
What are the traditional herbs that you consume, or have you ever been consumed?	Fenugreek	96	13.7
	Fennel	120	17.1
	What is written above	216	30.8
	Other	149	21.2
	I have never drunk traditional herbs	121	17.2
Question 3			
Have you ever consumed any source of phytoestrogen such as: soybean-soy milk and flaxseeds?	No	381	54.3
	Yes	321	45.7
Question 4			
What are the phytoestrogen sources that you consume or you have ever consumed?	Soybean-soy milk	122	17.4
	Flaxseeds	145	20.7
	Other	54	7.7
Question 5			
Have you stopped taking or drinking these herbs/plants?	Yes	243	34.6
	No	331	47.2
	I have never drunk them	128	18.2
Question 6			
How often do you or have you been consuming these herbs/plants?	Every day	44	6.3
	Every week	72	10.2
	During menstruation	288	41
	After childbirth (postpartum period)	52	7.4
	Between long intermittent periods	130	18.5
	Other times	107	15.2
	I have never consumed them	9	1.3
Question 7			
Since when did you start consuming traditional herbs/plants?	From one month to six months	63	8.9
	From six months to one year	40	5.7
	From one to two years	91	13
	Long period of time, for many years	386	55
	I have never consumed them	122	17.3
Question 8			
If you consume these herbs and plants, what is the purpose of consuming them?	To reduce pain and symptoms associated with menstruation	436	62.1
	To increase estrogen (female hormone)	33	4.7
	To lose weight	40	5.7
	Other reasons	77	11
	I have never used them	116	16.5

**Figure 1:**
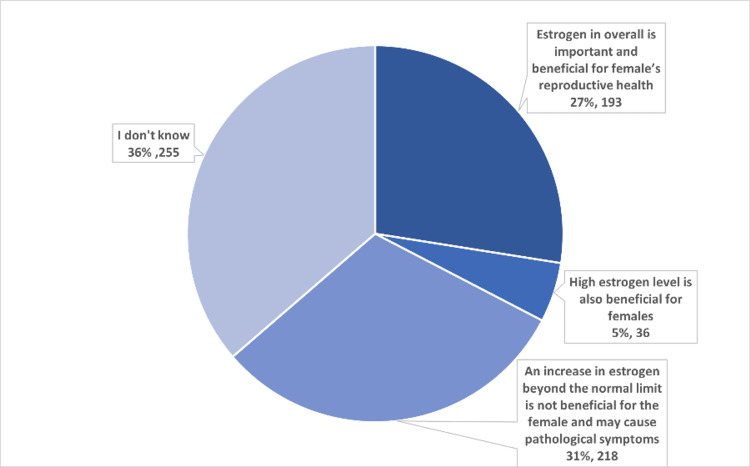
Knowledge of estrogen levels in the female body

Results regarding females’ knowledge about estrogen. In a total of 702 participants, most of them (n=255, 36.3%) reported that they did not know about the impact of high estrogen levels, as shown in Figure 1.

Regarding the association between the consumption of fenugreek and fennel and hormone-sensitive conditions. Those who consumed the herbs had a higher presence of different types of hormone-sensitive tumors and diseases. This positive relationship was found with a p-value of <0.001, as shown in Table 3.

**Table 3:** Association of hormone-sensitive conditions to the use of traditional herbs containing phytoestrogens (fenugreek and fennel)

Variables	Hormone-sensitive breast conditions			Hormone-sensitive uterine and ovarian conditions		
Use of traditional herbs	No	Yes	p-value	No	Yes	p-value
No (N=134)	127 (94.8%)	7 (5.2%)	<0.001	124 (92.5%)	10 (7.5%)	<0.001
Yes (N=568)	459 (80.8%)	109 (19.2%)		446 (78.5%)	122 (21.5%)	

About the relationship between the consumption of soy products and flaxseeds and hormone-sensitive conditions. Those who consumed soy products and flaxseeds had a higher presence of breast-related diseases; this positive association was found with a p-value of <0.001. Conversely, those who consumed soy products and flaxseeds had a lower presence of uterine and ovarian-related conditions; this negative association was found with a p-value of 0.368 which is greater than 0.001, as shown in Table 4.

**Table 4:** Association between breast, uterine, and ovarian health conditions, and the use of soy products and flaxseeds

Variables	Hormone-sensitive breast conditions			Hormone-sensitive uterine and ovarian conditions		
Use of traditional herbs	No	Yes	p-value	No	Yes	p-value
No (N=381)	347 (91.1%)	34 (8.9%)	<0.001	314 (82.4%)	67 (17.6%)	>0.001
Yes (N=321)	239 (74.5%)	82 (25.5%)		256 (79.8%)	65 (20.2%)	

**Figure 2:**
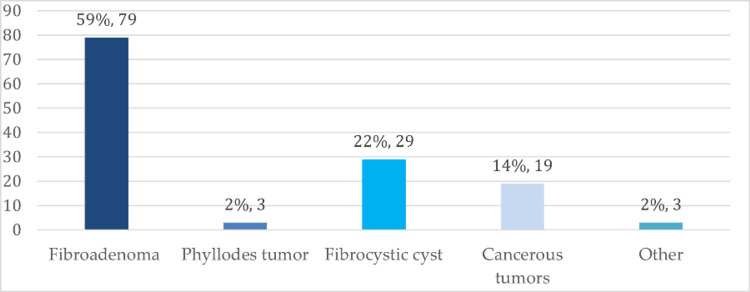
Frequency of breast-related health conditions (N=133)

For the study participants who had been asked certain questions related to the types of hormone-sensitive breast conditions, results indicated that most of the consumers with breast health issues (59.79%) had a fibroadenoma tumor, 22.29% had a fibrocystic cyst, and 14.19% had cancerous tumors, as shown in Figure 2.

**Figure 3:**
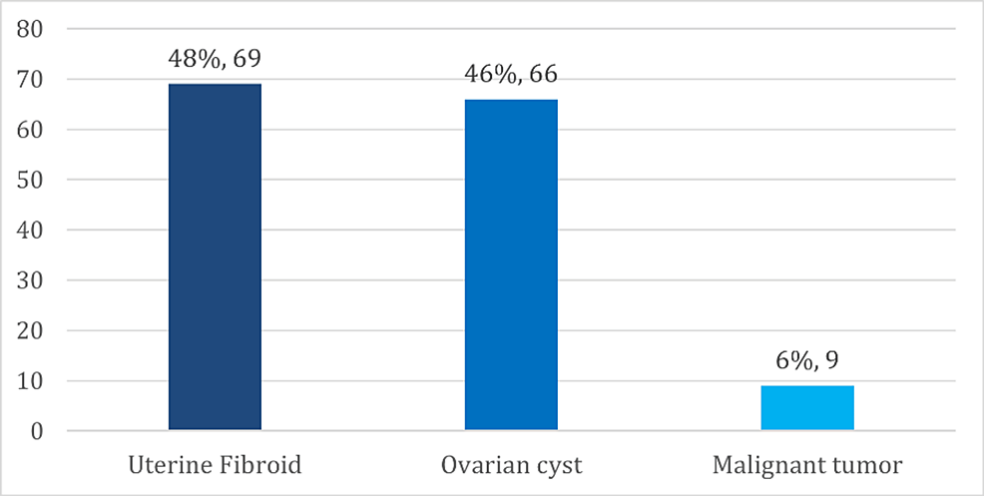
Frequency of uterine and ovarian-related health conditions (N=144)

For the types of hormone-sensitive uterine and ovarian conditions. Most of the consumers with uterine and ovarian health conditions reported that they had uterine fibroids (48.69%), and (46.66%) had ovarian cysts, as shown in Figure 3.

## Discussion

Most of the studies were interested in the beneficial role of phytoestrogens in cancer prevention and health [12], and there is a lack of medical studies investigating the association of traditional herbs containing phytoestrogens with estrogen-related health issues. This study may be one of the first studies conducted in Saudi Arabia for further investigation into this apparent gap in medical research. As the purpose of this study was to gain a better understanding of the impact of phytoestrogen consumption on the presence of hormone-dependent tumors and diseases, the data highlight a significant association between the presence of hormone-dependent tumors and diseases of the breast in females who consume traditional herbs containing phytoestrogens, such as fenugreek and fennel, with a statistically significant p-value of 0.000.

Moreover, another result that indicated the consumption of previously mentioned herbs could also contribute to a higher risk of uterine and ovarian-related diseases in females is significant, with a p-value of 0.000. These results were in harmony with an in vitro study done by Sreeja S et al. [13], which investigated the fenugreek effects and its capacity as an alternative to hormone replacement therapy and concluded that fenugreek stimulated the proliferation of MCF-7 breast cancer cells. In addition, Yancu D et al. [14] studied the effects of fennel seeds on the steroidogenesis of the tumor, which stimulated mammary tumor cell proliferation. 

In the present study, the results of participants showed that the presence of hormone-sensitive breast tumors and diseases was higher among consumers of soy products and flaxseed.

SaveThis positive relationship was significant with a p-value of 0.000, and this was in agreement with many experimental studies that support the potential risk of long-term exposure to soy-based foods containing phytoestrogens and its adverse outcomes related to breast cancer. According to the mechanism of action of estrogen mimics, the effect was observed in animal experimental studies [15].

Furthermore, a study done by McMichael-Phillips DF et al. [16] evaluated a group of premenopausal women who consumed 60g of soya supplementation (45mg isoflavones), and they found that this group of women had an increased number of breast epithelial cells histologically. Another pilot study done by Petrakis NL et al. [17] showed increased breast fluid secretion and the appearance of hyperplastic epithelial cells as well as increased levels of plasma estradiol. These results were associated with long-term consumption of a soy protein isolate with 38mg/d isoflavones. These studies raise concern that a diet rich in phytoestrogens could have stimulatory effects on a female’s health.

On the other hand, the available data of respondents stated that the presence of uterine and ovarian tumors and diseases is lower among soy-product and flaxseed consumers, with a p-value of 0.368. This result does not fit with a meta-analysis study that suggested that high soy-based food intake is associated with fibroids tumor growth [18].

In addition to that, a cohort study on African-American women supported by a questionnaire that was taken to evaluate the association between early life phytoestrogen exposure and increased uterine leiomyomas incidence in adulthood showed that the exposed women had significantly larger fibroids than the unexposed women [19], and this is in contrast with the proposed hypothesis. The available data on consuming soy products and flaxseed support the validity of previous studies that may promote the presence of breast tumors and diseases. However, for uterine and ovarian diseases, according to the data at hand, soy products and flaxseeds are not consumed a lot, and for a long time in our culture, in which the consumers of soy products and flaxseed represent a cumulative percentage of 38.1%. The percentage of consumers of fenugreek was 13.7%, and fennel was 17.1%, and for both of these two herbs, it was 30.8%, so the cumulative percentage was 61.6%. This gives the idea that the popularity of fenugreek and fennel is much higher in society than that of soy products and flaxseeds.

Moreover, some of the participants (n= 130, 18%) consume soy products and flaxseeds between long intermittent periods in their lives, unlike herbs such as fenugreek and fennel. In addition, genetic and environmental factors should be considered for the different consequences of these studies. The previous studies mentioned above focused on different ethnicities and cultural behaviors. Furthermore, the exact mechanism of phytoestrogen is still unknown and may perform differently depending on several environmental and physiological factors, such as the consumer’s reproductive age, bioavailability, and duration of consumption, in addition to the fact that these compounds are pharmacologically complicated [20].

In this study, we concluded that the most common types used were fenugreek and fennel, with a percentage of 30%. They use these herbs and plants containing phytoestrogens for extended periods (55%); this may be a factor contributing to the occurrence of female hormone-sensitive tumors and diseases. Concerning the participants, the intention of their consumption is mainly to find means to lessen the severity of menstrual symptoms (62.1%), and this may explain why certain herbs and plants are widely used in our culture.

Analysis of this study reported that the majority of participants (36%) had inadequate or poor knowledge about the impact of estrogen level elevation on female health. This result informs us that it is necessary to increase education regarding herbs and plants containing phytoestrogens and raise awareness through public health campaigns.

Limitations

Due to some limitations encountered in this study, we used a digital survey, which is a self-administered report rather than an official medical report. Other factors, such as contraceptive pill consumption or family history of tumors, genetic factors, and hormonal disorders, were not considered in our study. Moreover, there is a lack of previous similar studies to compare with our study results. In addition to that, there are contradictory responses in this data: 116 women said they don’t have breast tumors in the yes or no question, but when asked about the types, 133 women responded with different types.

Despite these limitations, the present study has enhanced our understanding of the relationship between consuming plants and herbs containing estrogen-mimetic substances and the presence of hormone-sensitive diseases and tumors.

## Conclusions

This study yielded important findings, as the consumption of fenugreek and fennel showed a potential detrimental association regarding the occurrence of hormonally sensitive tumors and diseases of the breast, uterus, and ovaries. However, breast diseases were more common among people who consumed flaxseeds and soy products, while uterine and ovarian diseases were less common. Regarding some limitations in our study, further large-scale studies are needed to investigate the effects of long-duration consumption of phytoestrogen herbs and plants on women’s health in consideration of the co-founding factors.

## Appendices

Appendix

Table 5 contains the study questionnaire.

**Table 5:** Study questionnaire

Informed consent: Participation is voluntary and all answers will be treated confidentially and are for research purposes.	
I agree to participate in this study:	Yes
	No
I promise that all the data are correct:	Yes
	No
Social and demographic characteristics	
Gender:	Female
Age:	From 18-25 years old
	From 26-35 years old
	From 36-45 years old
	From 46-55 years old
	From 56-65 years old
	Over 65 years old
Nationality:	Saudi
	Non-Saudi
Which Region:	Central Region
	Northern Region
	Eastern Region
	Western Region
	Southern Region
Social status:	Single
	Married
	Divorced
	Widow
Questions about consumption, beliefs, diseaes and tumors	
Have you ever consumed these herbs such as: fennel, fenugreek?	Yes
	No
Have you stopped taking or drinking these herbs/plants?	Yes
	No
	I have never drunk them
What are the traditional herbs that you consume, or have you ever been consumed?	Fenugreek
	Fennel
	Everything mentioned above
	Other
	I have never drunk traditional herbs
If you consume these herbs and plants, what is the purpose of consuming them?	Reducing the pain associated with menstruation
	To increase estrogen (female hormone)
	Weight loss
	Other reasons
	I have never used it
How often do you or have you been consuming these herbs/plants?	Everyday
	Every week
	Drink it during period time (menstruation)
	Drink it after childbirth (postpartum period)
	Between long intermittent periods
	I have never drunk traditional herbs
Since when did you start consuming traditional herbs?	A short period from one month to six months
	From six months to a year
	From one to two years
	Before a long period, two years or more
	I have never drunk traditional herbs
Do you take some sources of phytoestrogen such as: soybean or flaxseeds?	Soybean- soy-milk
	Flaxseeds
	I have never consumed other sources
	Other
Do you know that estrogen comes from other sources rather than herbs and plants?	Yes
	No
	I don't know
What do you think about the increase in estrogen levels (female hormone) in females?	Estrogen in overall is important and beneficial for female's reproductive health
	High estrogen level is also beneficial for females
	An increase in estrogen beyond the normal limit is not beneficial for the female and mau cause pathological symptoms
	I don't know
In your opinion, what can appear on a female because of a higher estrogen levels?	Weight gain
	Breast swelling and pain or the appearance of fibrous lumps or cysts
	Fibroids in the uterus
	Irregular menstruation or heavy bleeding
	Increasing symptoms of premenstrual syndrome
	Decrease sexual desire
	Hair loss
	General fatigue and exhaustion
	Increased estrogen does not cause anything and is beneficial to female health
	I don't know
Have you ever been pregnant or had a baby?	Yes
	No
If you answered yes to the previous question, how many times did you give birth?	Once
	Twice
	Three or more times
	I did not
Have you been diagnosed with a breast health problem?	Yes
	No
If yes, what breast health problem have you been diagnosed with?	Fibroadenoma tumor
	Phyllodes tumor
	Breast cyst
	Cancerous tumors
	I have not been diagnosed with breast tumors
Have you been diagnosed with a uterine or ovarian-related health problem?	Yes
	No
If yes, what condition in uterus or ovaries have you been diagnosed with?	Uterine fibroid
	Ovarian cysts
	Malignant tumor
	I have not been diagnosed with uterine tumors
